# 5-[(*E*)-Benzyl­idene]-2-hy­droxy-10-methyl-8-phenyl-3,10-diazahexa­cyclo­[10.7.1.1^3,7^.0^2,11^.0^7,11^.0^16,20^]henicosa-1(19),12(20),13,15,17-pentaen-6-one ethanol 0.25-solvate 0.6-hydrate

**DOI:** 10.1107/S1600536810039310

**Published:** 2010-10-09

**Authors:** Raju Suresh Kumar, Hasnah Osman, Subbu Perumal, Madhukar Hemamalini, Hoong-Kun Fun

**Affiliations:** a School of Chemical Sciences, Universiti Sains Malaysia, 11800 USM, Penang, Malaysia; b Department of Organic Chemistry, School of Chemistry, Madurai Kamaraj University, Madurai 625 021, India; c X-ray Crystallography Unit, School of Physics, Universiti Sains Malaysia, 11800 USM, Penang, Malaysia

## Abstract

In the title compound, C_33_H_28_N_2_O_2_·0.25C_2_H_6_O·0.6H_2_O, the piperidone ring adopts a chair conformation and the pyrrolidine ring adopts an envelope conformation. The dihedral angle between the two phenyl rings is 70.83 (16)°. One of the N atoms of the organic mol­ecule is disordered over two positions in a 0.52 (4):0.48 (4) ratio and the two solvent mol­ecules are partially occupied and show high displacement parameters. In the crystal, mol­ecules are connected by inter­molecular O—H⋯O and C—H⋯O hydrogen bonds, forming a three-dimensional network.

## Related literature

For details of 1,3-dipolar cyclo­addition reactions, see: Lown (1984 ▶); Tsuge & Kanemasa (1989 ▶); Williams & Fegley (1992 ▶); Periyasami *et al.* (2009 ▶); Suresh Babu & Raghunathan (2007 ▶). For puckering parameters, see: Cremer & Pople (1975 ▶). For the stability of the temperature controller used in the data collection, see: Cosier & Glazer (1986 ▶).
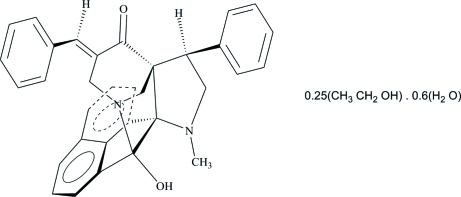


## Experimental

### 

#### Crystal data

C_33_H_28_N_2_O_2_·0.25C_2_H_6_O·0.6H_2_O
*M* *_r_* = 506.90
Tetragonal, 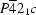

*a* = 19.3839 (3) Å
*c* = 14.0757 (2) Å
*V* = 5288.74 (14) Å^3^
*Z* = 8
Mo *K*α radiation
μ = 0.08 mm^−1^
*T* = 100 K
0.19 × 0.18 × 0.15 mm

#### Data collection

Bruker SMART APEXII CCD diffractometer
Absorption correction: multi-scan (*SADABS*; Bruker, 2009 ▶) *T*_min_ = 0.985, *T*_max_ = 0.988
23186 measured reflections
4230 independent reflections
3388 reflections with *I* > 2σ(*I*)
*R*_int_ = 0.041

#### Refinement

*R*[*F* ^2^ > 2σ(*F* ^2^)] = 0.058
*wR*(*F* ^2^) = 0.148
*S* = 1.03
4230 reflections
387 parameters
H atoms treated by a mixture of independent and constrained refinement
Δρ_max_ = 0.35 e Å^−3^
Δρ_min_ = −0.21 e Å^−3^

### 

Data collection: *APEX2* (Bruker, 2009 ▶); cell refinement: *SAINT* (Bruker, 2009 ▶); data reduction: *SAINT*; program(s) used to solve structure: *SHELXTL* (Sheldrick, 2008 ▶); program(s) used to refine structure: *SHELXTL*; molecular graphics: *SHELXTL*; software used to prepare material for publication: *SHELXTL* and *PLATON* (Spek, 2009 ▶).

## Supplementary Material

Crystal structure: contains datablocks global, I. DOI: 10.1107/S1600536810039310/hb5631sup1.cif

Structure factors: contains datablocks I. DOI: 10.1107/S1600536810039310/hb5631Isup2.hkl

Additional supplementary materials:  crystallographic information; 3D view; checkCIF report

## Figures and Tables

**Table 1:** Hydrogen-bond geometry (Å, °)

*D*—H⋯*A*	*D*—H	H⋯*A*	*D*⋯*A*	*D*—H⋯*A*
O1—H1*O*1⋯O1^i^	0.66 (4)	2.48 (4)	3.117 (5)	164 (5)
C17—H17*A*⋯O2^ii^	0.93	2.57	3.287 (6)	134

Symmetry codes: (i) 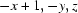; (ii) 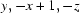.

**Fig. 1.**
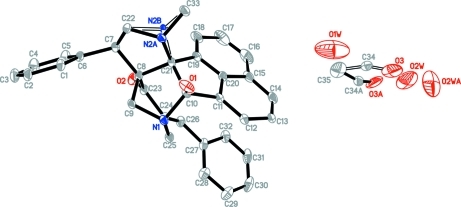
The asymmetric unit of (I), showing 30% probability displacement ellipsoids (H atoms are omitted for clarity). All disorder components are shown.

**Fig. 2.**
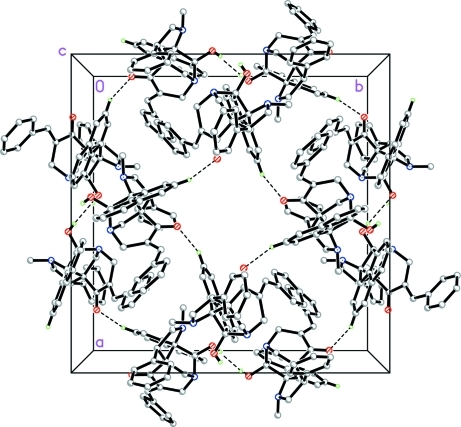
The crystal packing of (I), showing hydrogen-bonded (dashed lines) network. Solvent molecules, minor disorder components and H atoms are not involing the hydrogen bond interactions are omitted for clarity.

## supplementary crystallographic information

### Comment

Intermolecular 1,3-dipolar cycloadditions are one of the most useful processes for the construction of five-membered heterocycles containing the pyrrolidine structural unit (Lown, 1984; Tsuge *et al.*, 1989). This method is widely used for the synthesis of natural products such as alkaloids and pharmacologically important compounds (Williams *et al.*, 1992). 1,3-Dipolar cycloaddition of azomethine ylides with definite dipolarophiles provides a way for the synthesis of many dispiroheterocyclic systems (Periyasami *et al.*, 2009; Suresh Babu & Raghunathan, 2007). In view of their biological importance, herein we present the results of the crystal structure determination of the title compound.

The molecular structure of the title compound is shown in Fig. 1. The piperidone (N1/C8/C9/C23–C25) ring adopts a chair conformation [Q = 0.610 (3) Å, θ = 37.6 (3)°, φ = 57.8 (4)° ; Cremer & Pople, 1975]. The pyrrolidine ring N atom disordered over two sites with a refined occupancy ratio of 0.52 (4):0.48 (4).  The major (C7/C8/C21/C22/N2A) and minor (C7/C8/C21/C22/N2B) disordered pyrrolidine rings adopt the same envelope conformation with puckering parameters Q(2) = 0.349 (5) Å, φ = 73.0 (15)° for major disordered component and Q(2) = 0.459 (6) Å, φ = 332.4 (10) for minor disordered component.  The dihedral angle between the two phenyl rings (C1–C6 and C27–C32) is 70.80 (16)°.

In the crystal packing (Fig. 2), molecules are connected by intermolecular O1—H1O1···O1 and C17—H17A···O2 (Table 1) hydrogen bonds to form a three-dimensional network.

### Experimental

A mixture of 3,5-bis[(E)-phenylmethylidene]tetrahydro-4(1*H*)-pyridinone (0.100 g, 0.364 mmol), acenaphthenequinone (0.066 g, 0.364 mmol), and sarcosine (0.032 g, 0.272 mmol) were dissolved in methanol (5 mL) and refluxed for 1 hour. After completion of the reaction as evident from TLC, the mixture was poured into water (50 mL). The precipitated solid was filtered, washed with water and recrystallised from pet.ether-ethyl acetate mixture (1:1) to reveal pale yellow blocks of (I).

### Refinement

Anomalous dispersion was negligible and Friedel pairs were merged before refinement. Atom H1O1 was located from a difference Fourier map and refined freely. The remaining H atoms were positioned geometrically [O–H = 0.66 (4)–1.1395 Å and C–H = 0.93–0.97 Å] and were refined using a riding model, with *U*_iso_(H) = 1.2 or 1.5*U*_eq_(C, O). A rotating group model was applied to the methyl groups. In the final refinement, the occupancies of the water molecules were fixed at 40% whereas the occupancies of the atoms of the disorder ethanol molecule were fixed at 25%. The identities of the disordered and partially occupied solvent molecules are less certain; a PLATON SQUEEZE analysis indicated an electron count close to that modelled here.

### Crystal data

**Table:**

C_33_H_28_N_2_O_2_·0.25C_2_H_6_O·0.6H_2_O	*D*_x_ = 1.273 Mg m^−^^3^
*M**_r_* = 506.90	Mo *K*α radiation, λ = 0.71073 Å
Tetragonal, *P*42_1_*c*	Cell parameters from 5795 reflections
Hall symbol: P -4 2n	θ = 2.6–29.9°
*a* = 19.3839 (3) Å	µ = 0.08 mm^−^^1^
*c* = 14.0757 (2) Å	*T* = 100 K
*V* = 5288.74 (14) Å^3^	Block, pale yellow
*Z* = 8	0.19 × 0.18 × 0.15 mm
*F*(000) = 2148	

### Data collection

**Table:**

Bruker SMART APEXII CCD diffractometer	4230 independent reflections
Radiation source: fine-focus sealed tube	3388 reflections with *I* > 2σ(*I*)
graphite	*R*_int_ = 0.041
φ and ω scans	θ_max_ = 30.1°, θ_min_ = 1.5°
Absorption correction: multi-scan (*SADABS*; Bruker, 2009)	*h* = −23→24
*T*_min_ = 0.985, *T*_max_ = 0.988	*k* = −8→27
23186 measured reflections	*l* = −17→19

### Refinement

**Table:**

Refinement on *F*^2^	Primary atom site location: structure-invariant direct methods
Least-squares matrix: full	Secondary atom site location: difference Fourier map
*R*[*F*^2^ > 2σ(*F*^2^)] = 0.058	Hydrogen site location: inferred from neighbouring sites
*wR*(*F*^2^) = 0.148	H atoms treated by a mixture of independent and constrained refinement
*S* = 1.03	*w* = 1/[σ^2^(*F*_o_^2^) + (0.0762*P*)^2^ + 1.6944*P*] where *P* = (*F*_o_^2^ + 2*F*_c_^2^)/3
4230 reflections	(Δ/σ)_max_ < 0.001
387 parameters	Δρ_max_ = 0.35 e Å^−^^3^
0 restraints	Δρ_min_ = −0.21 e Å^−^^3^

### Special details

**Table:**

Experimental. The crystal was placed in the cold stream of an Oxford Cryosystems Cobra open-flow nitrogen cryostat (Cosier & Glazer, 1986) operating at 100.0 (1) K.
Geometry. All s.u.'s (except the s.u. in the dihedral angle between two l.s. planes) are estimated using the full covariance matrix. The cell s.u.'s are taken into account individually in the estimation of s.u.'s in distances, angles and torsion angles; correlations between s.u.'s in cell parameters are only used when they are defined by crystal symmetry. An approximate (isotropic) treatment of cell s.u.'s is used for estimating s.u.'s involving l.s. planes.
Refinement. Refinement of F^2^ against ALL reflections. The weighted R-factor wR and goodness of fit S are based on F^2^, conventional R-factors R are based on F, with F set to zero for negative F^2^. The threshold expression of F^2^ > 2σ(F^2^) is used only for calculating R-factors(gt) etc. and is not relevant to the choice of reflections for refinement. R-factors based on F^2^ are statistically about twice as large as those based on F, and R- factors based on ALL data will be even larger.

### Fractional atomic coordinates and isotropic or equivalent isotropic displacement parameters (Å^2^)

**Table:**

	*x*	*y*	*z*	*U*_iso_*/*U*_eq_	Occ. (<1)
O1	0.44856 (11)	0.06178 (12)	−0.00668 (19)	0.0410 (6)	
O2	0.54102 (12)	0.33051 (11)	−0.07672 (15)	0.0416 (5)	
N1	0.55166 (11)	0.12176 (11)	−0.02818 (15)	0.0242 (4)	
N2A	0.3846 (4)	0.1607 (11)	−0.0740 (5)	0.029 (3)	0.52 (4)
N2B	0.3748 (4)	0.1934 (11)	−0.0633 (5)	0.025 (3)	0.48 (4)
C1	0.48583 (15)	0.19294 (15)	−0.33651 (18)	0.0302 (6)	
H1A	0.4671	0.1504	−0.3200	0.036*	
C2	0.51981 (16)	0.20036 (17)	−0.42318 (19)	0.0364 (7)	
H2A	0.5236	0.1628	−0.4639	0.044*	
C3	0.54775 (18)	0.26274 (19)	−0.4490 (2)	0.0442 (8)	
H3A	0.5709	0.2674	−0.5065	0.053*	
C4	0.5409 (2)	0.3187 (2)	−0.3882 (2)	0.0526 (9)	
H4A	0.5592	0.3613	−0.4054	0.063*	
C5	0.50702 (19)	0.31165 (17)	−0.3015 (2)	0.0421 (8)	
H5A	0.5027	0.3496	−0.2615	0.050*	
C6	0.47963 (14)	0.24860 (15)	−0.27427 (18)	0.0277 (6)	
C7	0.44519 (14)	0.24214 (15)	−0.17726 (18)	0.0284 (6)	
H7A	0.4283	0.2879	−0.1591	0.034*	
C8	0.49319 (14)	0.21656 (14)	−0.09800 (17)	0.0262 (5)	
C9	0.53559 (14)	0.15141 (15)	−0.12184 (17)	0.0268 (5)	
H9A	0.5088	0.1194	−0.1599	0.032*	
H9B	0.5774	0.1633	−0.1559	0.032*	
C10	0.48373 (13)	0.12174 (14)	0.02031 (19)	0.0260 (5)	
C11	0.48822 (14)	0.13202 (16)	0.12660 (18)	0.0292 (6)	
C12	0.51434 (18)	0.0923 (2)	0.1980 (2)	0.0499 (9)	
H12A	0.5325	0.0489	0.1852	0.060*	
C13	0.51320 (18)	0.1187 (3)	0.2928 (2)	0.0662 (14)	
H13A	0.5295	0.0912	0.3421	0.079*	
C14	0.48900 (18)	0.1825 (3)	0.3127 (2)	0.0621 (13)	
H14A	0.4901	0.1984	0.3751	0.075*	
C15	0.46233 (16)	0.2252 (2)	0.2412 (2)	0.0434 (8)	
C16	0.4376 (2)	0.2932 (2)	0.2503 (3)	0.0632 (12)	
H16A	0.4376	0.3143	0.3096	0.076*	
C17	0.4135 (2)	0.3288 (2)	0.1732 (3)	0.0681 (13)	
H17A	0.3979	0.3737	0.1812	0.082*	
C18	0.41177 (19)	0.29897 (19)	0.0814 (3)	0.0497 (9)	
H18A	0.3945	0.3237	0.0300	0.060*	
C19	0.43589 (15)	0.23323 (16)	0.0697 (2)	0.0314 (6)	
C20	0.46115 (14)	0.19742 (16)	0.14849 (18)	0.0300 (6)	
C21	0.44354 (14)	0.18916 (15)	−0.01883 (18)	0.0271 (5)	
C22	0.38450 (14)	0.19137 (16)	−0.16933 (18)	0.0298 (6)	
H22A	0.3413	0.2154	−0.1802	0.036*	
H22B	0.3890	0.1555	−0.2169	0.036*	
C23	0.54231 (15)	0.26973 (15)	−0.05562 (19)	0.0294 (6)	
C24	0.59090 (14)	0.24127 (14)	0.01748 (19)	0.0284 (5)	
C25	0.60533 (13)	0.16417 (14)	0.01602 (19)	0.0265 (5)	
H25A	0.6483	0.1564	−0.0176	0.032*	
H25B	0.6118	0.1486	0.0809	0.032*	
C26	0.61200 (15)	0.28449 (15)	0.0862 (2)	0.0316 (6)	
H26A	0.5984	0.3303	0.0798	0.038*	
C27	0.65392 (16)	0.26835 (16)	0.17032 (19)	0.0335 (6)	
C28	0.70345 (16)	0.21629 (16)	0.1728 (2)	0.0375 (7)	
H28A	0.7123	0.1908	0.1181	0.045*	
C29	0.73987 (19)	0.20185 (18)	0.2554 (3)	0.0470 (9)	
H29A	0.7719	0.1662	0.2561	0.056*	
C30	0.7285 (2)	0.2406 (2)	0.3369 (3)	0.0536 (10)	
H30A	0.7526	0.2307	0.3924	0.064*	
C31	0.6811 (2)	0.2939 (2)	0.3350 (2)	0.0508 (10)	
H31A	0.6741	0.3207	0.3890	0.061*	
C32	0.64365 (17)	0.30786 (18)	0.2528 (2)	0.0393 (7)	
H32A	0.6116	0.3436	0.2524	0.047*	
C33	0.31851 (14)	0.15983 (19)	−0.0249 (2)	0.0404 (7)	
H33A	0.3259	0.1550	0.0422	0.061*	
H33B	0.3011	0.1173	−0.0502	0.061*	
H33C	0.2857	0.1960	−0.0362	0.061*	
O3	0.4685 (12)	0.459 (2)	0.8576 (13)	0.22 (2)	0.25
H1O3	0.4747	0.4920	0.9015	0.330*	0.25
C34	0.4750 (7)	0.4887 (8)	0.7781 (11)	0.046 (3)	0.25
H34A	0.4975	0.5271	0.8080	0.055*	0.25
H34B	0.4290	0.5052	0.7671	0.055*	0.25
C35	0.5000	0.5000	0.6848 (14)	0.097 (6)	0.50
H35A	0.4911	0.5467	0.6656	0.145*	0.25
H35B	0.5488	0.4916	0.6841	0.145*	0.25
H35C	0.4776	0.4689	0.6416	0.145*	0.25
O1W	0.4432 (8)	0.4776 (9)	0.629 (2)	0.270 (14)	0.40
H1W1	0.4418	0.4711	0.5691	0.405*	0.40
H2W1	0.4181	0.4646	0.6761	0.405*	0.40
O2W	0.5000	0.5000	0.9514 (16)	0.199 (13)	0.40
H1W2	0.5293	0.5433	0.9884	0.298*	0.40
H1O1	0.468 (2)	0.035 (2)	−0.017 (3)	0.068 (16)*	

### Atomic displacement parameters (Å^2^)

**Table:**

	*U*^11^	*U*^22^	*U*^33^	*U*^12^	*U*^13^	*U*^23^
O1	0.0307 (11)	0.0319 (11)	0.0604 (15)	−0.0074 (9)	0.0121 (11)	−0.0197 (11)
O2	0.0553 (14)	0.0358 (11)	0.0336 (11)	−0.0011 (10)	−0.0114 (10)	−0.0023 (9)
N1	0.0237 (10)	0.0298 (11)	0.0190 (10)	−0.0030 (8)	0.0026 (8)	−0.0070 (8)
N2A	0.018 (2)	0.048 (8)	0.021 (2)	0.000 (3)	0.0007 (18)	−0.009 (3)
N2B	0.027 (3)	0.040 (7)	0.009 (2)	0.005 (3)	0.0024 (19)	−0.007 (3)
C1	0.0362 (15)	0.0344 (14)	0.0200 (11)	−0.0052 (12)	0.0008 (11)	−0.0015 (11)
C2	0.0403 (16)	0.0482 (17)	0.0209 (12)	−0.0027 (14)	−0.0006 (11)	−0.0063 (13)
C3	0.0462 (18)	0.060 (2)	0.0263 (13)	−0.0122 (16)	0.0040 (13)	0.0040 (14)
C4	0.069 (2)	0.051 (2)	0.0378 (17)	−0.0256 (19)	0.0058 (17)	0.0069 (15)
C5	0.059 (2)	0.0383 (17)	0.0290 (14)	−0.0090 (15)	0.0011 (14)	−0.0021 (13)
C6	0.0293 (13)	0.0344 (14)	0.0193 (11)	−0.0014 (11)	−0.0038 (10)	−0.0013 (11)
C7	0.0307 (13)	0.0357 (14)	0.0187 (11)	0.0024 (11)	0.0007 (10)	−0.0061 (11)
C8	0.0297 (13)	0.0333 (14)	0.0157 (10)	−0.0008 (11)	−0.0011 (10)	−0.0047 (10)
C9	0.0243 (12)	0.0395 (14)	0.0165 (11)	−0.0004 (11)	0.0025 (9)	−0.0071 (10)
C10	0.0265 (12)	0.0277 (12)	0.0237 (11)	−0.0002 (10)	0.0042 (10)	−0.0054 (11)
C11	0.0240 (12)	0.0414 (15)	0.0223 (11)	0.0022 (11)	0.0072 (10)	0.0011 (11)
C12	0.0389 (18)	0.073 (2)	0.0373 (16)	0.0196 (18)	0.0158 (14)	0.0229 (17)
C13	0.0286 (17)	0.142 (4)	0.0275 (15)	0.022 (2)	0.0098 (13)	0.030 (2)
C14	0.0296 (16)	0.141 (4)	0.0155 (12)	0.004 (2)	0.0011 (12)	−0.007 (2)
C15	0.0315 (15)	0.073 (2)	0.0254 (14)	−0.0031 (15)	0.0050 (12)	−0.0205 (15)
C16	0.061 (2)	0.080 (3)	0.049 (2)	−0.005 (2)	0.019 (2)	−0.041 (2)
C17	0.074 (3)	0.049 (2)	0.081 (3)	0.011 (2)	0.033 (3)	−0.033 (2)
C18	0.0487 (19)	0.0463 (19)	0.054 (2)	0.0187 (16)	0.0187 (17)	−0.0037 (17)
C19	0.0309 (13)	0.0386 (15)	0.0246 (13)	0.0063 (11)	0.0069 (11)	−0.0057 (11)
C20	0.0282 (13)	0.0411 (15)	0.0208 (12)	0.0005 (12)	0.0009 (10)	−0.0090 (11)
C21	0.0274 (12)	0.0368 (14)	0.0172 (10)	0.0037 (11)	0.0007 (10)	−0.0053 (11)
C22	0.0296 (13)	0.0415 (15)	0.0183 (11)	0.0030 (12)	−0.0026 (10)	−0.0027 (11)
C23	0.0317 (14)	0.0368 (15)	0.0196 (11)	−0.0014 (11)	−0.0018 (10)	−0.0048 (11)
C24	0.0306 (13)	0.0342 (14)	0.0204 (11)	−0.0058 (11)	−0.0015 (11)	−0.0033 (11)
C25	0.0274 (12)	0.0316 (13)	0.0204 (11)	−0.0038 (10)	−0.0005 (10)	−0.0048 (10)
C26	0.0353 (14)	0.0341 (14)	0.0255 (12)	−0.0071 (12)	−0.0003 (11)	−0.0021 (11)
C27	0.0392 (16)	0.0364 (15)	0.0247 (13)	−0.0181 (12)	−0.0049 (12)	−0.0007 (11)
C28	0.0391 (16)	0.0344 (15)	0.0389 (16)	−0.0152 (12)	−0.0100 (14)	−0.0003 (13)
C29	0.0495 (19)	0.0395 (17)	0.0520 (19)	−0.0211 (15)	−0.0214 (17)	0.0140 (16)
C30	0.064 (2)	0.062 (2)	0.0350 (16)	−0.034 (2)	−0.0179 (16)	0.0168 (16)
C31	0.062 (2)	0.068 (2)	0.0221 (13)	−0.030 (2)	−0.0047 (15)	−0.0038 (15)
C32	0.0433 (17)	0.0459 (18)	0.0288 (14)	−0.0172 (14)	−0.0016 (13)	−0.0051 (13)
C33	0.0262 (13)	0.070 (2)	0.0254 (13)	0.0016 (14)	0.0034 (11)	−0.0101 (15)
O3	0.17 (2)	0.42 (6)	0.063 (9)	0.21 (3)	0.005 (11)	−0.045 (17)
C34	0.045 (8)	0.045 (8)	0.048 (8)	0.008 (6)	−0.007 (6)	−0.004 (6)
C35	0.132 (16)	0.048 (8)	0.111 (14)	0.029 (10)	0.000	0.000
O1W	0.118 (12)	0.157 (16)	0.54 (4)	−0.033 (11)	−0.04 (2)	0.00 (2)
O2W	0.25 (3)	0.19 (2)	0.16 (2)	0.16 (2)	0.000	0.000

### Geometric parameters (Å, °)

**Table:**

O1—C10	1.400 (3)	C17—H17A	0.9300
O1—H1O1	0.66 (4)	C18—C19	1.367 (4)
O2—C23	1.215 (4)	C18—H18A	0.9300
N1—C25	1.465 (3)	C19—C20	1.397 (4)
N1—C9	1.472 (3)	C19—C21	1.518 (4)
N1—C10	1.483 (3)	C22—H22A	0.9700
N2A—C33	1.456 (9)	C22—H22B	0.9700
N2A—C22	1.469 (7)	C23—C24	1.500 (4)
N2A—C21	1.487 (8)	C24—C26	1.344 (4)
N2B—C33	1.381 (7)	C24—C25	1.521 (4)
N2B—C21	1.474 (8)	C25—H25A	0.9700
N2B—C22	1.505 (8)	C25—H25B	0.9700
C1—C2	1.394 (4)	C26—C27	1.470 (4)
C1—C6	1.395 (4)	C26—H26A	0.9300
C1—H1A	0.9300	C27—C28	1.393 (5)
C2—C3	1.374 (5)	C27—C32	1.404 (4)
C2—H2A	0.9300	C28—C29	1.389 (4)
C3—C4	1.389 (5)	C28—H28A	0.9300
C3—H3A	0.9300	C29—C30	1.389 (6)
C4—C5	1.393 (5)	C29—H29A	0.9300
C4—H4A	0.9300	C30—C31	1.384 (6)
C5—C6	1.386 (4)	C30—H30A	0.9300
C5—H5A	0.9300	C31—C32	1.393 (5)
C6—C7	1.525 (4)	C31—H31A	0.9300
C7—C8	1.535 (4)	C32—H32A	0.9300
C7—C22	1.538 (4)	C33—H33A	0.9600
C7—H7A	0.9800	C33—H33B	0.9598
C8—C23	1.525 (4)	C33—H33C	0.9601
C8—C9	1.544 (4)	O3—C34	1.27 (3)
C8—C21	1.565 (4)	O3—C34^i^	1.87 (3)
C9—H9A	0.9700	O3—H1O3	0.9000
C9—H9B	0.9700	C34—C34^i^	1.06 (3)
C10—C11	1.512 (4)	C34—C35	1.42 (2)
C10—C21	1.618 (4)	C34—O3^i^	1.87 (3)
C11—C12	1.363 (4)	C34—H34A	0.9596
C11—C20	1.406 (4)	C34—H34B	0.9599
C12—C13	1.430 (6)	C35—C34^i^	1.42 (2)
C12—H12A	0.9300	C35—H35A	0.9600
C13—C14	1.353 (7)	C35—H35B	0.9600
C13—H13A	0.9300	C35—H35C	0.9600
C14—C15	1.401 (6)	O1W—H35C	0.7091
C14—H14A	0.9300	O1W—H1W1	0.8579
C15—C16	1.410 (6)	O1W—H2W1	0.8566
C15—C20	1.412 (4)	O2W—O2W^ii^	1.37 (5)
C16—C17	1.367 (6)	O2W—H1O3	0.8695
C16—H16A	0.9300	O2W—H1W2	1.1395
C17—C18	1.415 (6)		
			
C10—O1—H1O1	116 (5)	C19—C21—C10	102.8 (2)
C25—N1—C9	108.2 (2)	C8—C21—C10	102.7 (2)
C25—N1—C10	115.81 (19)	N2A—C22—N2B	26.4 (2)
C9—N1—C10	103.0 (2)	N2A—C22—C7	109.0 (4)
C33—N2A—C22	115.9 (8)	N2B—C22—C7	98.7 (4)
C33—N2A—C21	115.6 (9)	N2A—C22—H22A	109.9
C22—N2A—C21	109.1 (6)	N2B—C22—H22A	92.1
C33—N2B—C21	121.5 (7)	C7—C22—H22A	109.9
C33—N2B—C22	118.3 (7)	N2A—C22—H22B	109.9
C21—N2B—C22	107.8 (7)	N2B—C22—H22B	135.7
C2—C1—C6	120.7 (3)	C7—C22—H22B	109.9
C2—C1—H1A	119.7	H22A—C22—H22B	108.3
C6—C1—H1A	119.7	O2—C23—C24	122.5 (2)
C3—C2—C1	120.6 (3)	O2—C23—C8	123.2 (3)
C3—C2—H2A	119.7	C24—C23—C8	114.3 (2)
C1—C2—H2A	119.7	C26—C24—C23	117.1 (3)
C2—C3—C4	119.1 (3)	C26—C24—C25	124.5 (3)
C2—C3—H3A	120.4	C23—C24—C25	117.9 (2)
C4—C3—H3A	120.4	N1—C25—C24	115.3 (2)
C3—C4—C5	120.6 (3)	N1—C25—H25A	108.5
C3—C4—H4A	119.7	C24—C25—H25A	108.5
C5—C4—H4A	119.7	N1—C25—H25B	108.5
C6—C5—C4	120.6 (3)	C24—C25—H25B	108.5
C6—C5—H5A	119.7	H25A—C25—H25B	107.5
C4—C5—H5A	119.7	C24—C26—C27	128.0 (3)
C5—C6—C1	118.4 (3)	C24—C26—H26A	116.0
C5—C6—C7	119.2 (3)	C27—C26—H26A	116.0
C1—C6—C7	122.4 (3)	C28—C27—C32	118.2 (3)
C6—C7—C8	114.3 (2)	C28—C27—C26	123.7 (3)
C6—C7—C22	116.9 (2)	C32—C27—C26	118.1 (3)
C8—C7—C22	101.8 (2)	C29—C28—C27	121.1 (3)
C6—C7—H7A	107.8	C29—C28—H28A	119.4
C8—C7—H7A	107.8	C27—C28—H28A	119.4
C22—C7—H7A	107.8	C30—C29—C28	120.1 (4)
C23—C8—C7	116.4 (2)	C30—C29—H29A	119.9
C23—C8—C9	107.8 (2)	C28—C29—H29A	119.9
C7—C8—C9	115.4 (2)	C31—C30—C29	119.6 (3)
C23—C8—C21	109.6 (2)	C31—C30—H30A	120.2
C7—C8—C21	104.7 (2)	C29—C30—H30A	120.2
C9—C8—C21	101.8 (2)	C30—C31—C32	120.5 (3)
N1—C9—C8	103.74 (19)	C30—C31—H31A	119.8
N1—C9—H9A	111.0	C32—C31—H31A	119.8
C8—C9—H9A	111.0	C31—C32—C27	120.5 (3)
N1—C9—H9B	111.0	C31—C32—H32A	119.8
C8—C9—H9B	111.0	C27—C32—H32A	119.8
H9A—C9—H9B	109.0	N2B—C33—N2A	27.6 (3)
O1—C10—N1	107.9 (2)	N2B—C33—H33A	108.3
O1—C10—C11	114.0 (2)	N2A—C33—H33A	109.7
N1—C10—C11	113.8 (2)	N2B—C33—H33B	122.5
O1—C10—C21	110.1 (2)	N2A—C33—H33B	98.2
N1—C10—C21	105.7 (2)	H33A—C33—H33B	109.5
C11—C10—C21	105.0 (2)	N2B—C33—H33C	96.6
C12—C11—C20	119.1 (3)	N2A—C33—H33C	119.8
C12—C11—C10	132.5 (3)	H33A—C33—H33C	109.5
C20—C11—C10	108.3 (2)	H33B—C33—H33C	109.5
C11—C12—C13	118.7 (4)	C34—O3—C34^i^	33.2 (13)
C11—C12—H12A	120.6	C34—O3—H1O3	105.4
C13—C12—H12A	120.6	C34^i^—O3—H1O3	86.8
C14—C13—C12	121.7 (3)	C34^i^—C34—O3	106.1 (16)
C14—C13—H13A	119.1	C34^i^—C34—C35	67.9 (7)
C12—C13—H13A	119.1	O3—C34—C35	157.4 (16)
C13—C14—C15	121.3 (3)	C34^i^—C34—O3^i^	40.7 (9)
C13—C14—H14A	119.4	O3—C34—O3^i^	77 (2)
C15—C14—H14A	119.4	C35—C34—O3^i^	105.7 (12)
C14—C15—C16	127.8 (3)	C34^i^—C34—H34A	42.8
C14—C15—C20	116.4 (3)	O3—C34—H34A	90.7
C16—C15—C20	115.8 (3)	C35—C34—H34A	97.5
C17—C16—C15	121.0 (3)	O3^i^—C34—H34A	17.8
C17—C16—H16A	119.5	C34^i^—C34—H34B	135.3
C15—C16—H16A	119.5	O3—C34—H34B	101.7
C16—C17—C18	121.8 (3)	C35—C34—H34B	96.8
C16—C17—H17A	119.1	O3^i^—C34—H34B	117.4
C18—C17—H17A	119.1	H34A—C34—H34B	103.7
C19—C18—C17	118.8 (4)	C34—C35—C34^i^	44.1 (13)
C19—C18—H18A	120.6	C34—C35—H35A	110.1
C17—C18—H18A	120.6	C34^i^—C35—H35A	100.2
C18—C19—C20	119.1 (3)	C34—C35—H35B	108.7
C18—C19—C21	131.1 (3)	C34^i^—C35—H35B	72.4
C20—C19—C21	109.7 (2)	H35A—C35—H35B	109.5
C19—C20—C11	113.9 (2)	C34—C35—H35C	109.6
C19—C20—C15	123.4 (3)	C34^i^—C35—H35C	147.0
C11—C20—C15	122.7 (3)	H35A—C35—H35C	109.5
N2B—C21—N2A	26.5 (2)	H35B—C35—H35C	109.5
N2B—C21—C19	103.2 (7)	H35C—O1W—H1W1	103.6
N2A—C21—C19	124.2 (7)	H35C—O1W—H2W1	106.1
N2B—C21—C8	103.6 (3)	H1W1—O1W—H2W1	134.2
N2A—C21—C8	103.1 (3)	O2W^ii^—O2W—H1O3	143.8
C19—C21—C8	117.0 (2)	O2W^ii^—O2W—H1W2	62.8
N2B—C21—C10	128.7 (8)	H1O3—O2W—H1W2	141.4
N2A—C21—C10	104.3 (8)		
			
C6—C1—C2—C3	−0.1 (5)	C18—C19—C21—N2A	−69.1 (8)
C1—C2—C3—C4	−0.9 (5)	C20—C19—C21—N2A	113.4 (7)
C2—C3—C4—C5	0.8 (6)	C18—C19—C21—C8	61.8 (4)
C3—C4—C5—C6	0.2 (6)	C20—C19—C21—C8	−115.8 (3)
C4—C5—C6—C1	−1.1 (5)	C18—C19—C21—C10	173.4 (3)
C4—C5—C6—C7	177.7 (3)	C20—C19—C21—C10	−4.1 (3)
C2—C1—C6—C5	1.1 (4)	C23—C8—C21—N2B	132.1 (9)
C2—C1—C6—C7	−177.7 (3)	C7—C8—C21—N2B	6.6 (9)
C5—C6—C7—C8	−93.8 (3)	C9—C8—C21—N2B	−114.0 (9)
C1—C6—C7—C8	85.0 (3)	C23—C8—C21—N2A	159.4 (9)
C5—C6—C7—C22	147.5 (3)	C7—C8—C21—N2A	33.8 (9)
C1—C6—C7—C22	−33.7 (4)	C9—C8—C21—N2A	−86.7 (9)
C6—C7—C8—C23	78.6 (3)	C23—C8—C21—C19	19.4 (3)
C22—C7—C8—C23	−154.5 (2)	C7—C8—C21—C19	−106.2 (3)
C6—C7—C8—C9	−49.3 (3)	C9—C8—C21—C19	133.3 (2)
C22—C7—C8—C9	77.7 (3)	C23—C8—C21—C10	−92.4 (2)
C6—C7—C8—C21	−160.3 (2)	C7—C8—C21—C10	142.1 (2)
C22—C7—C8—C21	−33.3 (3)	C9—C8—C21—C10	21.5 (2)
C25—N1—C9—C8	−74.5 (2)	O1—C10—C21—N2B	9.4 (6)
C10—N1—C9—C8	48.6 (2)	N1—C10—C21—N2B	125.7 (6)
C23—C8—C9—N1	71.6 (2)	C11—C10—C21—N2B	−113.7 (6)
C7—C8—C9—N1	−156.3 (2)	O1—C10—C21—N2A	−2.6 (5)
C21—C8—C9—N1	−43.6 (2)	N1—C10—C21—N2A	113.7 (4)
C25—N1—C10—O1	−157.8 (2)	C11—C10—C21—N2A	−125.6 (4)
C9—N1—C10—O1	84.4 (3)	O1—C10—C21—C19	128.2 (2)
C25—N1—C10—C11	−30.3 (3)	N1—C10—C21—C19	−115.5 (2)
C9—N1—C10—C11	−148.1 (2)	C11—C10—C21—C19	5.1 (3)
C25—N1—C10—C21	84.4 (2)	O1—C10—C21—C8	−109.9 (2)
C9—N1—C10—C21	−33.4 (2)	N1—C10—C21—C8	6.4 (2)
O1—C10—C11—C12	57.4 (4)	C11—C10—C21—C8	127.0 (2)
N1—C10—C11—C12	−67.0 (4)	C33—N2A—C22—N2B	61.8 (13)
C21—C10—C11—C12	178.0 (3)	C21—N2A—C22—N2B	−70.8 (14)
O1—C10—C11—C20	−125.1 (3)	C33—N2A—C22—C7	132.3 (10)
N1—C10—C11—C20	110.5 (3)	C21—N2A—C22—C7	−0.3 (14)
C21—C10—C11—C20	−4.6 (3)	C33—N2B—C22—N2A	−71.7 (15)
C20—C11—C12—C13	0.0 (5)	C21—N2B—C22—N2A	71.1 (13)
C10—C11—C12—C13	177.3 (3)	C33—N2B—C22—C7	172.7 (13)
C11—C12—C13—C14	−2.1 (6)	C21—N2B—C22—C7	−44.5 (12)
C12—C13—C14—C15	1.6 (6)	C6—C7—C22—N2A	146.6 (9)
C13—C14—C15—C16	−177.7 (4)	C8—C7—C22—N2A	21.3 (9)
C13—C14—C15—C20	1.0 (5)	C6—C7—C22—N2B	171.7 (8)
C14—C15—C16—C17	179.4 (4)	C8—C7—C22—N2B	46.4 (8)
C20—C15—C16—C17	0.7 (6)	C7—C8—C23—O2	5.6 (4)
C15—C16—C17—C18	0.5 (7)	C9—C8—C23—O2	137.1 (3)
C16—C17—C18—C19	−1.0 (6)	C21—C8—C23—O2	−112.9 (3)
C17—C18—C19—C20	0.3 (5)	C7—C8—C23—C24	−176.6 (2)
C17—C18—C19—C21	−177.1 (3)	C9—C8—C23—C24	−45.1 (3)
C18—C19—C20—C11	−176.4 (3)	C21—C8—C23—C24	64.8 (3)
C21—C19—C20—C11	1.5 (4)	O2—C23—C24—C26	27.3 (4)
C18—C19—C20—C15	1.0 (5)	C8—C23—C24—C26	−150.5 (3)
C21—C19—C20—C15	178.9 (3)	O2—C23—C24—C25	−160.3 (3)
C12—C11—C20—C19	−180.0 (3)	C8—C23—C24—C25	21.9 (3)
C10—C11—C20—C19	2.2 (3)	C9—N1—C25—C24	50.7 (3)
C12—C11—C20—C15	2.6 (5)	C10—N1—C25—C24	−64.2 (3)
C10—C11—C20—C15	−175.3 (3)	C26—C24—C25—N1	147.8 (3)
C14—C15—C20—C19	179.7 (3)	C23—C24—C25—N1	−24.0 (4)
C16—C15—C20—C19	−1.5 (5)	C23—C24—C26—C27	173.9 (3)
C14—C15—C20—C11	−3.2 (5)	C25—C24—C26—C27	2.1 (5)
C16—C15—C20—C11	175.7 (3)	C24—C26—C27—C28	30.3 (5)
C33—N2B—C21—N2A	73.1 (17)	C24—C26—C27—C32	−150.0 (3)
C22—N2B—C21—N2A	−68.3 (12)	C32—C27—C28—C29	2.5 (4)
C33—N2B—C21—C19	−72.3 (16)	C26—C27—C28—C29	−177.8 (3)
C22—N2B—C21—C19	146.3 (10)	C27—C28—C29—C30	−1.6 (5)
C33—N2B—C21—C8	165.3 (13)	C28—C29—C30—C31	−0.5 (5)
C22—N2B—C21—C8	23.9 (13)	C29—C30—C31—C32	1.6 (5)
C33—N2B—C21—C10	46.4 (18)	C30—C31—C32—C27	−0.6 (5)
C22—N2B—C21—C10	−95.0 (8)	C28—C27—C32—C31	−1.5 (4)
C33—N2A—C21—N2B	−59.1 (13)	C26—C27—C32—C31	178.8 (3)
C22—N2A—C21—N2B	73.7 (13)	C21—N2B—C33—N2A	−70.6 (17)
C33—N2A—C21—C19	−17.1 (16)	C22—N2B—C33—N2A	66.9 (14)
C22—N2A—C21—C19	115.6 (8)	C22—N2A—C33—N2B	−67.3 (13)
C33—N2A—C21—C8	−153.3 (10)	C21—N2A—C33—N2B	62.2 (14)
C22—N2A—C21—C8	−20.5 (13)	C34^i^—O3—C34—C35	71 (5)
C33—N2A—C21—C10	99.6 (12)	C34^i^—O3—C34—O3^i^	−28.9 (19)
C22—N2A—C21—C10	−127.6 (11)	O3—C34—C35—C34^i^	−79 (5)
C18—C19—C21—N2B	−51.2 (7)	O3^i^—C34—C35—C34^i^	15.4 (12)
C20—C19—C21—N2B	131.2 (6)		

Symmetry codes: (i) −*x*+1, −*y*+1, *z*; (ii) *y*, −*x*+1, −*z*+2.

### Hydrogen-bond geometry (Å, °)

**Table:**

*D*—H···*A*	*D*—H	H···*A*	*D*···*A*	*D*—H···*A*
O1—H1O1···O1^iii^	0.66 (4)	2.48 (4)	3.117 (5)	164 (5)
C17—H17A···O2^iv^	0.93	2.57	3.287 (6)	134

Symmetry codes: (iii) −*x*+1, −*y*, *z*; (iv) *y*, −*x*+1, −*z*.
